# Comparative study of pancreatic vessels and mesopancreas of rhesus monkeys and humans

**DOI:** 10.3389/fsurg.2023.1112316

**Published:** 2023-06-02

**Authors:** Fan Ye, Hang Xiong, Hongyu Su, Ziheng Huang, Wenxin Luo, Dongmei Yuan, Tao Yi, Hongying Zhou

**Affiliations:** ^1^ Department of Human Anatomy, West China School of Basic Medical Sciences and Forensic Medicine, Sichuan University, Chengdu, China; ^2^ West China School of Stomatology, Sichuan University, Chengdu, China; ^3^ Biotherapy Laboratory of Gynecological Oncology, Key Laboratory of Obstetric and Gynecologic and Pediatric Diseases and Birth Defects of the Ministry of Education, West China Second Hospital, Sichuan University, Chengdu, China

**Keywords:** mesopancreas, comparative anatomy, monkey’s pancreatic vessels, rhesus monkeys, total mesopancreas excision (TMpE)

## Abstract

**Introduction:**

With the introduction of the concept of mesopancreas defining the perineural structures that includes neurovascular bundle and lymph nodes extending from the posterior surface of the pancreatic head to behind the mesenteric vessels,Total Mesopancreas Excision (TMpE) based on this theory has facilitated the development of pancreatic cancer surgery in clinical practice in recent years. However, the existence of so called mesopancreas in the human body is still in debate and the comparative study of mesopancreas of rhesus monkey and human have not been well investigated.

**Purpose:**

The aim of our study is to compare the pancreatic vessels and fascia of human and rhesus monkeys in anatomical and embryological perspectives and to support the utilization of rhesus monkey as animal model.

**Methods:**

In this study, 20 rhesus monkey cadavers were dissected and their mesopancreas location, relationships and arterial distribution were analyzed. We compared the location and developmental patterns of mesopancreas in macaques and humans.

**Results:**

The results showed that the distribution of pancreatic arteries in rhesus monkeys was the same as that in humans, which is consistent with phylogenetic similarities. However, the morphological features of the mesopancreas and greater omentum is anatomically different from that of humans, including (1) the greater omentum is not connected to the transverse colon in monkeys. (2) The presence of the dorsal mesopancreas of the rhesus monkey suggests that it be an intraperitoneal organ. Comparative anatomical studies of mesopancreas and arteries in macaques and humans showed characteristic patterns of mesopancreas and similarities in pancreatic artery development in nonhuman primates, consistent with phylogenetic differentiation.

## 1. Introduction

Non-human primate (NHPs) species have many biological characteristics similar to those of humans and have a wide range of applications in taxonomy, ecology, anatomy, genetics, behavior, evolution, reproductive biology, and other fields of study (1, 2). Pancreatic diseases have always been difficult to diagnose and treat because of its deep location, many surrounding adjacent organs and complex structure. While diabetes-inducing non-human primates (NHPs) can serve as an ideal model for this pancreatic disease due to their high similarity to diabetic patients in terms of metabolic, hormonal and pathological abnormalities (3). Zhang et al. reported four modeling methods in rhesus monkeys, including total and partial pancreatectomy, the latter is more effective but needs a fully comprehension of pancreas anatomy (4). And anatomical studies on the pancreatic arteries of rhesus monkeys are scarce so far.

In recent years, with the introduction of the concept of “mesopancreas”, defining the perineural structures that includes neurovascular bundle and lymph nodes extending from the posterior surface of the pancreatic head to behind the mesenteric vessels, there has been a great evolution in the way of pancreatic resection (5, 6). Accordingly, the concept of “Total mesopancreas excision (TMpE)” has been proposed and applied, suggesting that TMpE can help raise the R0 resection rate and improve the prognosis (7–12). Although the concept of TMpE has been upheld in clinical practice for surgeries, the existence or non-existence as well as boundaries and contents of the mesopancreas are disputed (13). Agrawal et al. revealed loose areolar tissue, adipose tissue, peripheral nerve, nerve plexus, lymphatics and capillaries in retropancreatic tissue from 20 fresh cadavers; but these structures were not surrounded by fibrous sheaths or fascia, similar to the mesentery (14). Meanwhile, the blood supply of the pancreaticoduodenal region is one of the most complicated parts of the abdominal cavity in humans (15, 16). The extent of clinical surgical resection and structural preservation in TMpE are also controversial, especially with regard to the state of existence of the mesopancreas in the corresponding tissues of the posterior part of the pancreas. Hence, a clear understanding of the coverage of the peripancreatic fascia/peritoneum and the relationship between the fascia and surrounding structures enables clinical staff to resolve questions in clinical practice from a morphological aspect (17).

The aim of our study is to compare the pancreatic vessels and fascia of human and rhesus monkeys and to provide the basis for the utilization of rhesus monkeys from anatomical and embryological perspectives. A comprehensive understanding of the features of the peripancreatic peritoneum in terms of gross observation and embryology, and the elucidation of the relationship with surrounding organs, blood vessels and nerves are of great importance for the improvement of surgical standards for TMpE based on the theory of mesopancreas, improving the R0 resection rate of malignant tumors and ensuring surgical outcomes.

## 2. Materials and methods

### 2.1. Specimen collection

Rhesus monkeys Cadavers (*n* = 20) were purchased from Hengshu Bio-technology (Sichuan, China) for this study. This study was approved by the Committee on Animal Researchand the ethics committee of the Sichuan University (Chengdu, China).

### 2.2. Anatomic procedure

The abdominal cavity was opened surgically following standard surgical procedure, Midline laparotomy was performed and the greater omentum was incised for exposing the surface of the pancreas. Then we followed the layers of the mesopancreas for anatomical observation.

## 3. Results

### 3.1. Anatomy of anterior relations and surface of mesopancreas

**Figure 1:**
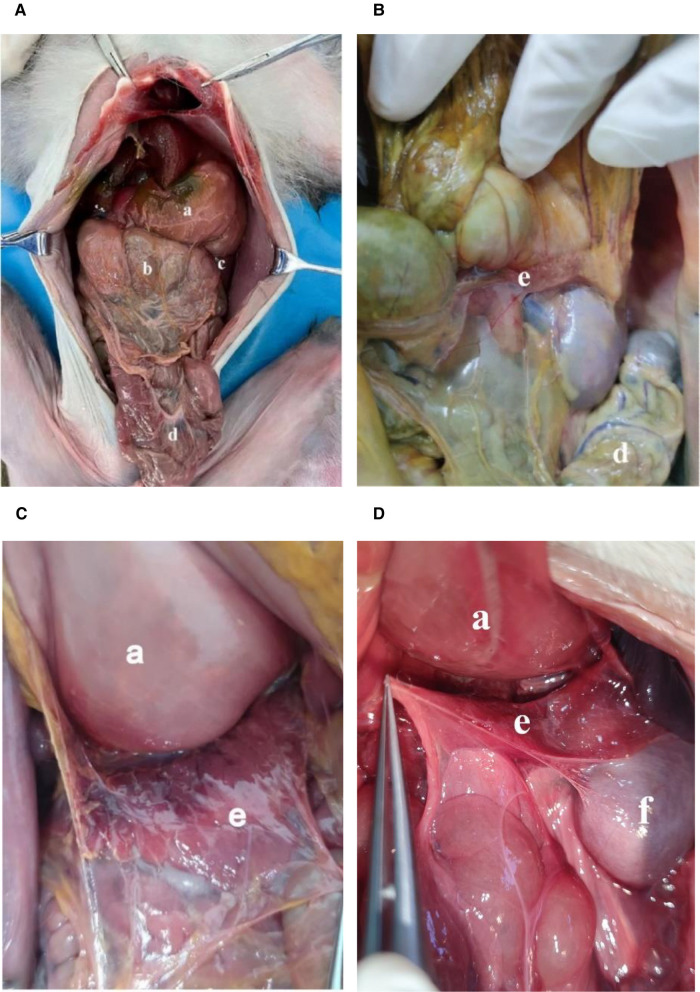
(A,B,C,D): Abdominal cavity and surface of mesopancreas of monkeys. (a): Stomach; (b): Greater omentum; (c): Spleen; (d): Transverse colon; (e): Pancreas; f: kidney.

The arrangement of peritoneal organs in monkeys is similar to the humans, shown in Figure 1A. The peritoneum on the anterior and posterior wall of the stomach is healed at the greater curvature of the stomach, forming the front of the greater omentum. But unlike in humans, the greater omentum in monkeys is not connected to the transverse colon. Instead, it goes directly backward to the greater curvature of the stomach. The transverse mesocolon in monkeys is attached to the pancreas and upward to the posterior abdominal wall (Figures 1B,C). The anterior layer of mesopancreas attached to the transverse mesentery is extended and fused posteriorly with the peritoneum of the posterior abdominal wall. It does not differ from that of humans (Figure 1D).

### 3.2. Anatomy of body and tail of mesopancreas

**Figure 2:**
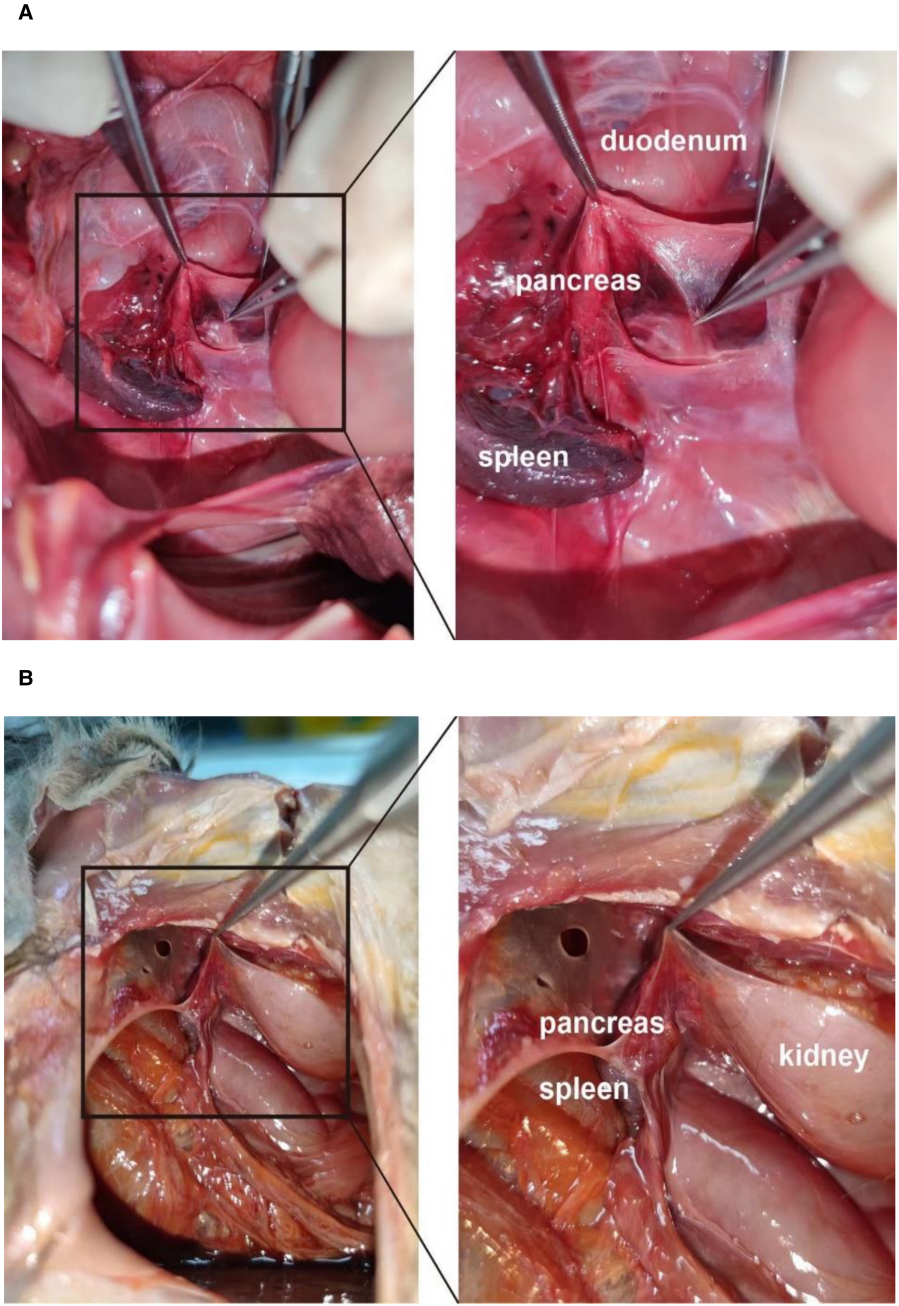
(A,B): Body and tail of the dorsal mesopancreas of monkeys.

The gastrosplenic ligament and short gastric artery of monkeys were cut and the peritoneum of the posterior abdominal wall above the pancreas was incised, observing that the pancreas was still attached to the posterior abdominal wall with fascia (Figure 2A). In humans, the dorsal aspect of the pancreas is located in the anterior pararenal space. In a lateral incision at the level of the second lumbar vertebra dorsally, the caudal pancreatic fascia was fused with the renal fascia and gastrosplenic ligament while attached to the posterior lateral abdominal wall in monkeys. The tail of the pancreas is also covered by the peritoneum (Figure 2B). This evidence suggests that the macaque pancreas is an intraperitoneal organ, in contrast to the view that the human pancreas is an extraperitoneal organ.

### 3.3. The arteries of the pancreas

**Figure 3:**
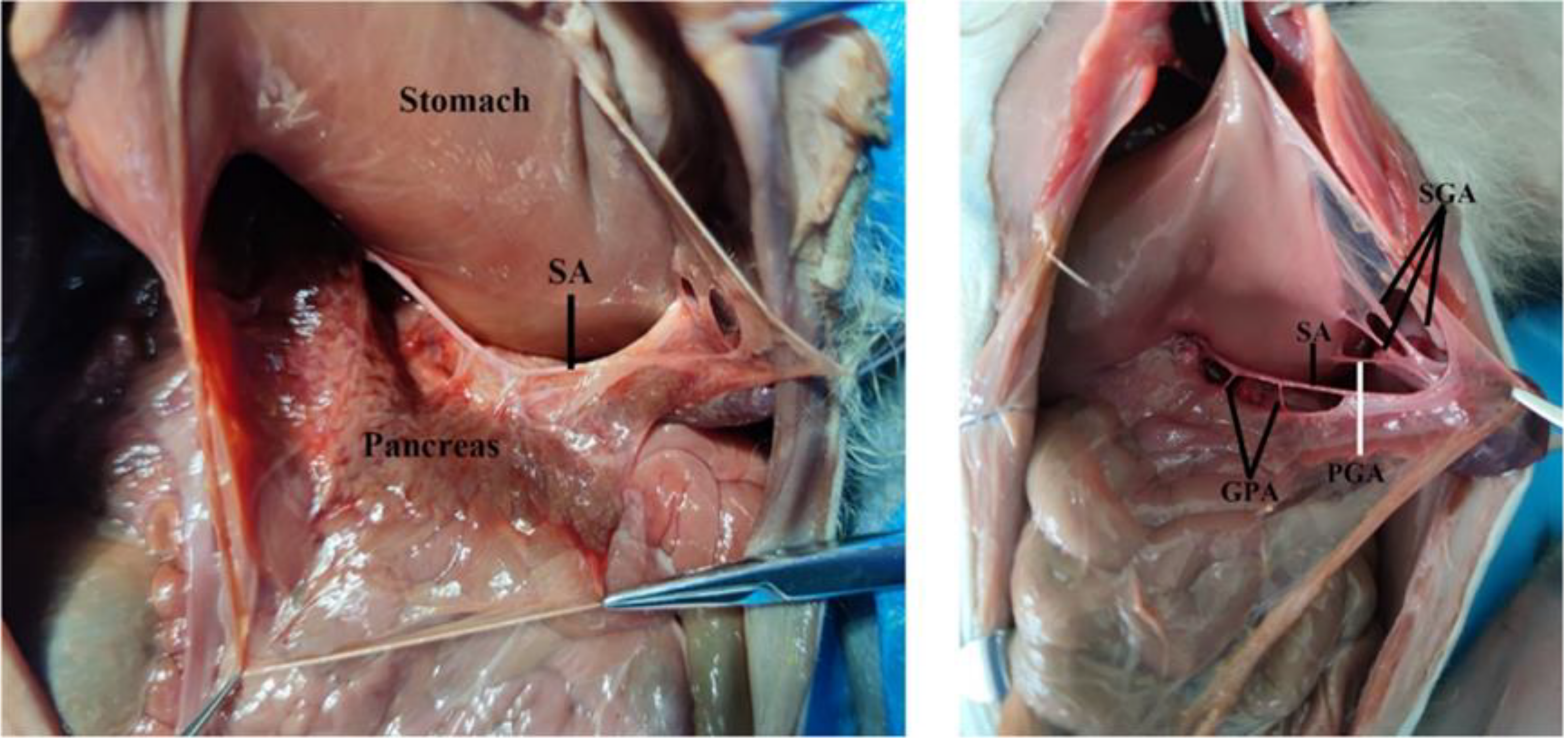
The arteries of the pancreas of monkeys. SA, splenic artery; SGA, short gastric artery; GPA, great pancreatic artery; PGA, posterior gastric artery.

The monkey's pancreas, posterior to the stomach, is an intraperitoneal organ. The greater omentum and the gastrocolic ligament were incised for exposing the pancreas and the splenic artery (Figure 3). The splenic artery (SA) forms a vascular arch at the superior margin of the pancreas within the mesopancreas, while the splenic artery in humans does not generally form a vascular arch. The pancreatic arteries mainly originate from the splenic artery. Adjacent to the splenic hilum, the splenic artery gives off the posterior gastric artery (PGA) and short gastric arteries (SGA). Arterial branching pattern of rhesus monkeys do not differ much from those of humans.

## 4. Discussion

**Figure 4:**
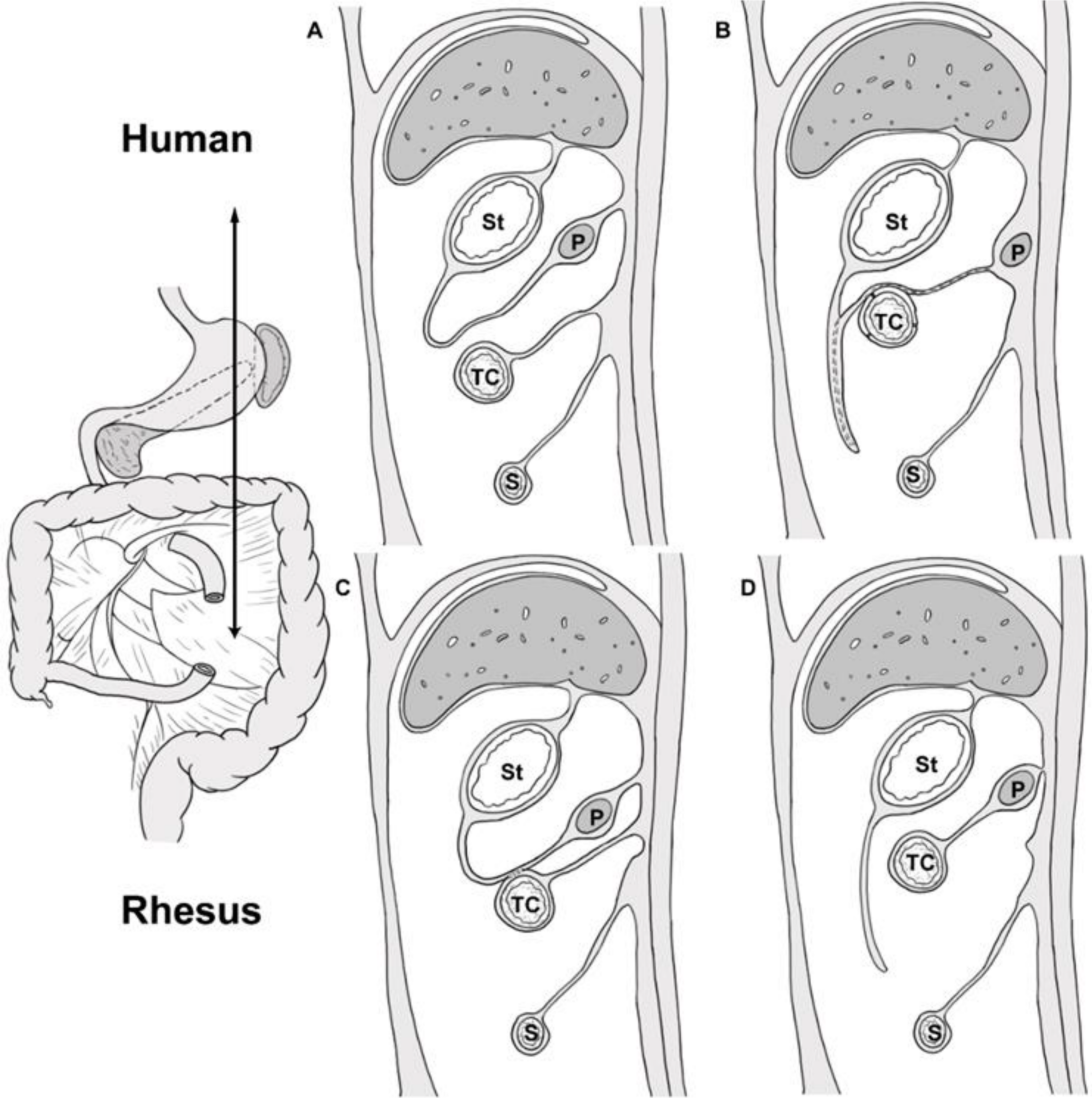
Schematic showing growth and development of greater omentum and mesopancreas in human and rhesus monkeys, sagittal sectional view at the level of the double arrow line. St, stomach; TC, transverse colon; P, pancreas; S, small bowel. (**A,B**) shows the fusion of the dorsal peritoneum of the omental sac with the transverse mesocolon at the rotation of stomach, and the disappearance of the dorsal mesentery of the pancreas. (**C,D**): the hypothetical sketch of the growth and development of the greater omentum and mesopancreatic organ in the rhesus monkey; The greater omentum and the transverse mesocolon may fuse and separate during the rotation of stomach in rhesus monkeys. Subsequently, the transverse mesocolon fused with the mesopancreas.

The comparative anatomy of humans and NHPs has a long history. Galen used monkeys to infer human anatomy. Tyson's 1699 work showing that chimps share many anatomical similarities with humans (18). NHPs are very suitable as animal models of embryonic development and anatomy.In this study, 20 rhesus monkey cadavers were dissected and their mesopancreas location relationships and arterial distribution were analyzed. The term “mesopancreas” (similar to mesorectum) has been controversial since its introduction by Gockel et al. (5, 14). The results of the study by Gockel et al. showed that the mesopancreas was a whitish-firm, fatty tissue-like layer locating dorsally to the pancreas and reaching beyond the mesenteric vessels (5). However, Sharma et al. deemed that due to not having a fascial envelope attaching the pancreas to the posterior wall of the abdomen and not containing “all” its blood vessels and “all” its primary draining lymphatics and lymph nodes, mesopancreas is a misnomer and cannot be called a “true” mesentery (19). We compared the location and developmental patterns of mesopancreas in macaques and humans. The results showed that the distribution of pancreatic arteries in rhesus monkeys was the same as that in humans, which is consistent with phylogenetic similarities. However, the development of the mesopancreas and greater omentum is anatomically different from that of humans and macaques, including (1) the greater omentum is not connected to the transverse colon in monkeys (2). The presence of the dorsal mesopancreas of the rhesus monkey suggests that it may be an intraperitoneal organ. Comparative anatomical studies of mesopancreas and arteries in macaques and humans showed characteristic patterns of mesopancreas development and similarities in pancreatic artery development in nonhuman primates, consistent with phylogenetic differentiation.The pancreas is traditionally viewed as an extraperitoneal organ, and yet the idea of mesopancreas is in conflict with traditional anatomy. However, we found that the dorsal mesopancreas in rhesus monkeys is intact and may be an animal model for studying the development of the mesopancreas. From an embryological perspective, the ventral pancreas and dorsal pancreas develop from the differentiation of the foregut during the 5th week of embryogenesis, and they are surrounded by primitive peritoneum like other organs. The ventral pancreas and dorsal pancreas gradually fuse with the peritoneum of the posterior abdominal wall as the stomach rotates and the pancreas grows and develops during the 6th week of embryonic life, which makes the pancreas an extraperitoneal organ (Figures 4A,B) (20). However, the greater omentum of the rhesus monkey is not connected to the transverse colon, a finding consistent with Zhang's study (4), and the dorsal mesopancreas is also present. We hypothesize that it is possible that the ventral greater omentum, the dorsal peritoneum of the omental sac, and the mesentery fused at the rotation of stomach. During subsequent development, the transverse mesocolon separated from the greater omentum and fused with the mesopancreas, detaching it from the development of the dorsal gastric mesentery and thus preserving the intact mesopancreas,we summarized this hypothesis in Figures 4C,D. This allowed the rhesus monkey pancreas to become an intraretroperitoneal organ. The importance of this unique development remains unclear. This study is the first to report this finding.

Despite controversy over the concept of the mesopancreas, the total mesopancreas excision (TMpE) based on this theory has facilitated the development of pancreatic cancer surgery in clinical practice (21–23). Yi et al. considered the mesopancreas is not a classical mesentery, such as the mesorectum, and it may be better termed the mesopancreatoduodenum than the mesopancreas (17). Sugiyama et al. designed a novel artery-first approach, in which the anterior mesopancreas was divided into the head and neck of the mesopancreas and the body and tail of the mesopancreas by using the abdominal aorta, superior mesenteric artery and midline of the celiac trunk as the dividing line. This operation ensures a good surgical field of view through a simplified anatomical approach and facilitates the rational removal of the mesopancreas during pancreaticoduodenectomy, improving the accuracy of the operation (24). However, current surgical criteria for TMpE are variable, study sample sizes are small and prospective randomized controlled studies with larger samples and long-term follow-up are lacking. More researches are still needed.

At present, the understanding of mesenteric development in the embryonic period is too little, and the precise morphological changes and mechanism of the evolution are still unknown (25). In addition, the researches on the pancreas of rhesus monkeys have focused on endocrine diseases, drug experiments and other fields, and less on its morphology and embryology (26, 27). With the rapid development of “membrane anatomy”, the understanding of mesenteric development will be improved, and rhesus macaques can be used as an accurate model system to study human Mesenteric development. In future work we will use techniques such as MRI or CT to analyze the detailed anatomical relationships around the pancreas of rhesus monkeys through 3D reconstruction.

## 5. Conclusion

In summary, we found differences in the mesopancreas between humans and rhesus monkeys. According to our results, the morphological features of the mesopancreas and greater omentum is anatomically different from that of humans and macaques and the differences between the two developmental processes are also compared, which indicates the phylogenetic uniqueness. We did not find differences between monkey pancreatic vasculature and human, which indicates the phylogenetic similarity between human and rhesus monkeys. Future studies with larger samples are needed to understand the growth and developmental processes of mesopancreas and greater omentum.

## Data Availability

The original contributions presented in the study are included in the article, further inquiries can be directed to the corresponding author.

## References

[1] Zhang XL Pang W Hu XT Li JL Yao YG Zheng YT. Experimental primates and non-human primate (nhp) models of human diseases in China: current Status and progress. Dongwuxue Yanjiu. (2014) 35(6):447–64. 10.13918/j.issn.2095-8137.2014.6.447 25465081 PMC4790274

[2] Pound LD Kievit P Grove KL. The nonhuman primate as a model for type 2 diabetes. Curr Opin Endocrinol Diabetes Obes. (2014) 21(2):89–94. 10.1097/med.0000000000000043 24569549

[3] King AJ. The use of animal models in diabetes research. Br J Pharmacol. (2012) 166(3):877–94. 10.1111/j.1476-5381.2012.01911.x 22352879 PMC3417415

[4] Zhang Y Fu L Lu YR Guo ZG Zhang ZD Cheng JQ Pancreas anatomy and surgical procedure for pancreatectomy in rhesus monkeys. J Med Primatol. (2011) 40(6):376–82. 10.1111/j.1600-0684.2011.00499.x 21895681

[5] Gockel I Domeyer M Wolloscheck T Konerding MA Junginger T. Resection of the mesopancreas (Rmp): a new surgical classification of a known anatomical space. World J Surg Oncol. (2007) 5(1):44. 10.1186/1477-7819-5-44 17459163 PMC1865381

[6] Morales E Zimmitti G Codignola C Manzoni A Garatti M Sega V Follow “the superior mesenteric artery”: laparoscopic approach for total mesopancreas excision during pancreaticoduodenectomy. Surg Endosc. (2019) 33(12):4186–91. 10.1007/s00464-019-06994-6 31332566

[7] Mahdi B Mohamed MM Mohamed FC Selim S Hassen T Hassen H Retroportal lamina or mesopancreas? Lessons learned by anatomical and histological study of thirty-three cadaveric dissections. Int J Surg. (2013) 11:834–6. 10.1016/2013-08-009 23994001

[8] Du F Wang X Lin H Zhao X. Pancreaticoduodenectomy with arterial approach of total mesenteric resection of the pancreas for pancreatic head cancer. Gastroenterology Res. (2019) 12(5):256–62. 10.14740/gr1225 31636776 PMC6785284

[9] Ramia JM De-la-Plaza R Manuel-Vazquez A Lopez-Marcano A Morales R. Systematic review of the mesopancreas: concept and clinical implications. Clin Transl Oncol. (2018) 20(11):1385–91. 10.1007/s12094-018-1869-5 29675778

[10] Adham M Singhirunnusorn J. Surgical technique and results of total mesopancreas excision (Tmpe) in pancreatic tumors. Eur J Surg Oncol. (2012) 38(4):340–5. 10.1016/j.ejso.2011.12.015 22264964

[11] Popescu I Dumitrascu T. Total meso-pancreas excision: key point of resection in pancreatic head adenocarcinoma. Hepatogastroenterology. (2011) 58(105):202–7. PMID: 21510315

[12] Xu J Tian X Chen Y Ma Y Liu C Tian L Total mesopancreas excision for the treatment of pancreatic head cancer. J Cancer. (2017) 8(17):3575–84. 10.7150/jca.21341 29151943 PMC5687173

[13] Eduardo SMF Oliver S Camila G Jose MAM-J Orlando JMT. What do surgeons need to know about the mesopancreas. Langenbeck Arch Surg. (2021) 406:2621–2632. 10.1007/s00423-021-02211-y 34117891

[14] Manish KA Dilip ST Uday S Shiv KC Dhananjaya S. Mesopancreas: myth or reality. JOP. (2010) 11(13):199–298. 10.6092/1590-8577/381820442512

[15] Wang HY Li MX Xiu DR. Shark mouth pancreaticojejunostomy: a new enteric reconstruction procedure of pancreatic stump. Chin Med J (Engl). (2019) 132(11):1354–8. 10.1097/CM9.0000000000000219 30896569 PMC6629351

[16] Wellner UF Brett S Bruckner T Limprecht R Rossion I Seiler C Pancreatogastrostomy versus pancreatojejunostomy for reconstruction after partial pancreatoduodenectomy (recopanc): study protocol of a randomized controlled trial Utn U1111-1117-9588. Trials. (2012) 13:45. 10.1186/1745-6215-13-45 22540372 PMC3478188

[17] Yi S Nagakawa Y Ren K Dai YD Zhang M Chen JF The mesopancreas and pancreatic head Plexus: morphological, developmental, and clinical perspectives. Surg Radiol Anat. (2020) 42(12):1501–8. 10.1007/s00276-020-02547-y 32797265

[18] Diogo R. Links between the discovery of primates and anatomical comparisons with humans, the chain of being, our place in nature, and racism. J Morphol. (2018) 279(4):472–93. 10.1002/jmor.20783 29194710

[19] Sharma D Isaji S. Mesopancreas is a misnomer: time to correct the nomenclature. J Hepatobiliary Pancreat Sci. (2016) 23(12):745–9. 10.1002/jhbp.402 27734589

[20] Standring S. Gray’s anatomy E-book: The anatomical basis of clinical practice. Amsterdam: Elsevier Health Sciences (2021).

[21] Maplanka C. A comprehensive study of the mesopancreas as an extension of the pancreatic circumferential resection margin. Eur Surg. (2018) 50(4):147–59. 10.1007/s10353-018-0535-z

[22] Wu W Wang X Wu X Li M Weng H Cao Y Total mesopancreas excision for pancreatic head cancer: analysis of 120 cases. Chin J Cancer Res. (2016) 28(4):423–8. 10.21147/j.issn.1000-9604.2016.04.05 27647970 PMC5018537

[23] Serrablo-Requejo A Paterna-López S Gutiérrez-Díez M Abadía-Forcén MT Serradilla-Martín M Sancho-Pardo P Impact of total mesopancreas excision on postoperative tumor recurrence. Int J Surg. (2022) 100:25–7. 10.1016/j.ijsu.2022.106559

[24] Sugiyama M Suzuki Y Nakazato T Yokoyama M Kogure M Matsuki R Vascular anatomy of mesopancreas in pancreatoduodenectomy using an intestinal derotation procedure. World J Surg. (2020) 44(10):3441–8. 10.1007/s00268-020-05605-z 32474625

[25] Ehrenpreis ED Alverdy JC Wexner SD. The mesenteric organ in health and disease. Berlin: Springer (2021).

[26] Tsuchitani M Sato J Kokoshima H. A comparison of the anatomical structure of the pancreas in experimental animals. J Toxicol Pathol. (2016) 29(3):147–54. 10.1293/tox.2016-0016 27559239 PMC4963614

[27] Wolfe-Coote S Louw J Woodroof C Du Toit DF. The non-human primate endocrine pancreas: development, regeneration potential and metaplasia. Cell Biol Int. (1996) 20(2):95–101. 10.1006/cbir.1996.0013 8935153

